# Insertion bias and purifying selection of retrotransposons in the *Arabidopsis thaliana *genome

**DOI:** 10.1186/gb-2004-5-10-r79

**Published:** 2004-09-29

**Authors:** Vini Pereira

**Affiliations:** 1 Imperial College London, Silwood Park Campus, Buckhurst Road, Ascot, Berkshire SL5 7PY, UK

## Abstract

**Background:**

Genome evolution and size variation in multicellular organisms are profoundly influenced by the activity of retrotransposons. In higher eukaryotes with compact genomes retrotransposons are found in lower copy numbers than in larger genomes, which could be due to either suppression of transposition or to elimination of insertions, and are non-randomly distributed along the chromosomes. The evolutionary mechanisms constraining retrotransposon copy number and chromosomal distribution are still poorly understood.

**Results:**

I investigated the evolutionary dynamics of long terminal repeat (LTR)-retrotransposons in the compact *Arabidopsis thaliana *genome, using an automated method for obtaining genome-wide, age and physical distribution profiles for different groups of elements, and then comparing the distributions of young and old insertions. Elements of the Pseudoviridae family insert randomly along the chromosomes and have been recently active, but insertions tend to be lost from euchromatic regions where they are less likely to fix, with a half-life estimated at approximately 470,000 years. In contrast, members of the Metaviridae (particularly *Athila*) preferentially target heterochromatin, and were more active in the past.

**Conclusion:**

Diverse evolutionary mechanisms have constrained both the copy number and chromosomal distribution of retrotransposons within a single genome. In *A. thaliana*, their non-random genomic distribution is due to both selection against insertions in euchromatin and preferential targeting of heterochromatin. Constant turnover of euchromatic insertions and a decline in activity for the elements that target heterochromatin have both limited the contribution of retrotransposon DNA to genome size expansion in *A. thaliana*.

## Abstract 2

An analysis of the distribution and age of LTR retrotransposons in the *Arabidopsis* genome revealed that Pseudoviridae insert randomly along the chromosome and have been recently active whereas Metaviridae were more active in the past and preferentially target heterochromatin.

## Background

It has become increasingly clear that the activity of transposable elements (TEs) is a major cause of genome evolution. TEs are ubiquitous components of eukaryotic genomes. For example, 22% of the *Drosophila melanogaster *[1], 45% of the human [2], and up to 80% of the maize [3] genomes consist of TE fossils. TEs have influenced the evolution of cellular gene regulation and function, and have been responsible for chromosomal rearrangements [4]. Variation in genome size and the C-value paradox [5] can be attributed to a large extent to differences in the amount of TEs, particularly of retrotransposons, between the genomes of different species [6]. In plant genomes, large size and structural variation even among closely related species is mainly due to differences in their history of polyploidization [7] and/or amplification of long terminal repeat (LTR)-retrotransposons [3,8-10]. LTR-retrotransposons (LTR-RTs) are 'copy-and-paste' (class I) TEs that replicate via an RNA intermediate. Like retroviruses, their (intact) genome consists of two LTRs, which contain the signals for transcription initiation and termination, flanking an internal region (IR) that typically contains genes and other features necessary for autonomous retrotransposition. LTR-RTs are mainly classified into two major families, the Pseudoviridae (also known as *Ty1/Copia *elements) and Metaviridae (*Ty3/Gypsy*).

The evolutionary forces that control copy number and shape the chromosomal distribution of different kinds of TEs in eukaryotic genomes are still poorly understood. Some large plant and animal genomes have expanded owing to an ability to tolerate massive amplification of retrotransposons, whereas in more compact genomes these elements are found in lower copy numbers, non-randomly distributed and mainly confined to heterochromatic regions [11-14]. TEs have mostly been regarded as parasitic DNA [15,16], and it has been suggested that important epigenetic mechanisms originally evolved to suppress the activity of TEs and other foreign genetic material [17]. Nevertheless, there are examples of individual elements that have been co-opted by, and entire TE families that have become mutualists to, their host genomes [13].

It is often hypothesized that the non-random genomic distribution of TEs in some species reflects the action of purifying selection on the host against the deleterious effects of TE insertions in certain regions. Models differ in the kind of deleterious effects they propose: chromosomal rearrangements due to 'ectopic' (unequal homologous) recombination [18]; disruption of gene regulation due to insertion near cellular genes [19]; or a burden on cell physiology as a result of the expression of TE-encoded products [20]. In compact genomes, clustering of TE insertions in silent heterochromatin, which has reduced rates of recombination, gene density and levels of transcription, is in principle consistent with a scenario of negative selection and of passive accumulation of TEs where their insertions would be less deleterious. As an alternative to purifying selection, another hypothesis to explain this clustering of TEs involves preferential insertion, or even positive selection for their retention, into heterochromatin [21].

To evaluate these hypotheses, I investigated the evolutionary history of different groups of LTR-RTs in the *Arabidopsis thaliana *genome. The total TE content of the compact genome of *A. thaliana*, with a haploid size of approximately 150 Mbp (million base-pairs), has been previously estimated as around 10%, and is known to cluster around the pericentromeric heterochromatin [14]. Despite the relatively low copy numbers, there is a high diversity of LTR-RTs in *A. thaliana *[22,23]. I have implemented an automated methodology for genome-wide sequence mining of LTR-RTs, and for estimating the age of insertion of different copies. This methodology is capable of identifying nested insertions, which are common in the pericentromeric regions. The technique for dating LTR-RTs has been previously used to reveal a massive amplification of these elements that doubled the size of the maize genome during the last 3 million years, by extrapolation of results found in a 240 kbp stretch of intergenic DNA [3]. Here I report genome-wide age profiles for different groups of LTR-RTs in *A. thaliana*. By comparing the age and chromosomal distributions of young and old insertions it is possible to distinguish between preferential targeting and passive accumulation of elements into heterochromatin. I show that members of the Pseudoviridae have recently been active, that they integrate randomly into the genome (relative to centromere location) and only passively accumulate in proximal regions, as purifying selection eliminates euchromatic insertions. In contrast, the Metaviridae (particularly members of the *Athila *group) preferentially insert into the pericentromeric heterochromatin, and their transpositional activity has declined in the last million years.

## Results

### Abundance and diversity

Most of the retrieved elements are fragmented and truncated, and nested insertions are common particularly among pericentromeric elements belonging to the *Athila *superfamily, though the core centromere sequences themselves were not available. In fact, the size of the *A. thaliana *genome has been recently estimated as approximately 157 Mbp (around 20% larger than the estimate published with the genome sequence), and the additional size appears to be due to (unsequenced) heterochromatic repetitive DNA in the centromeres, telomeres and nucleolar-organizing regions [24].  Table 1 shows the relative abundance of each superfamily, and the numbers of complete and solo-LTR elements identified in the genome. *Athila *is the most abundant superfamily, followed by the *Copia*-like, *Gypsy*-like, and TRIM (terminal-repeat retrotransposons in miniature). The ratio of solo-LTRs to complete elements is around 2:1. In addition to solo-LTR formation, deletion and fragmentation of retrotransposon DNA in *A. thaliana *also occur via other mechanisms: 36% of the DNA in the *Athila*, 38% in the *Gypsy*-like, 32% in the *Copia*-like, and 21% in the TRIM superfamilies correspond to degraded insertions that are neither 'complete' elements nor solo-LTRs.

### Age distribution

To obtain the genome-wide age distribution of each superfamily (except TRIM), 564 pairs of intra-element LTRs were (pairwise) aligned and their sequence divergence estimated. Many of the complete TRIM elements have highly divergent LTRs, and I suspect that extensive recombination between inter-element LTRs has occurred. In neighbor-joining trees of LTR sequences (of both complete and solo elements) from the TRIM families *Katydid-At1 *and *Katydid-At2*, most intra-element LTR pairs did not cluster. In contrast, when trees were constructed for representatives of the *Athila *(*athila2*), *Gypsy*-like (*atlantys2*), and *Copia*-like (*meta1*, *atcopia49*, *atcopia78*) superfamilies, intra-element LTR pairs always clustered (data not shown), providing evidence for the lack of inter-element recombination in those 'families'.

**Figure 1:**
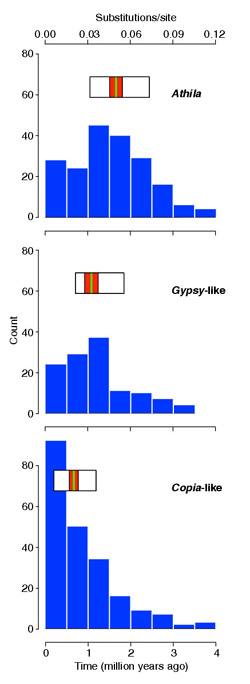
Age distributions of LTR-retrotransposon superfamilies. *Athila *insertions are on average significantly older, and *Copia*-like ones younger, than those from other superfamilies. There are 34 *Copia*-like, four *Athila*, and three *Gypsy*-like insertions with identical intra-element LTRs. The width of the horizontal boxes above the histograms indicates the middle 50% of age values in each superfamily; the red band indicates 95% confidence limits on the median, and the green stripe the median value.

The superfamilies differ significantly in their average age of insertions. *Athila *insertions are significantly older than the *Gypsy*-like (Wilcoxon rank-sum test, *p *< 0.0005), *Gypsy*-like older than *Copia*-like (*p *< 0.0001). Age distributions are summarized in Figure 1.

### *Copia*-like insertions are younger than host species

Using the rate of 1.5 × 10^-8 ^substitutions per site per year [25], 97% of 215 complete *Copia*-like elements are younger than 3 million years (Myr), 90% younger than 2 Myr, and only two insertions estimated to be older than 4 Myr. This shows that complete insertions from the known *Copia*-like families in the *A. thaliana *genome are younger than the species itself, whose time of divergence from its closest relatives, such as *A. lyrata *has been estimated (with the same rate of evolution) to be 5.1-5.4 Myr ago [25]. The situation is less clear for *Athila *(and the *Gypsy*-like TEs), as 7% of 219 intra-element LTR pairs were estimated to be older than 5 Myr (3% of the *Gypsy*-like). Furthermore, the *Athila *and *Gypsy*-like superfamilies have an excess of degraded insertions relative to *Copia*-like (Table 1). Complete elements account for around 50% of the total amount of DNA in *Athila *and *Gypsy*-like, indicating that the majority of insertions remaining in the genome have been degraded or have become solo-LTRs. Some of these are likely to be older than the complete insertions. DNA loss (from LTR-RTs) has been shown to occur in *A. thaliana *[26], and the oldest insertions may have been degraded beyond detection. On the other hand, there is some evidence that synonymous sites in *Arabidopsis *are not evolving in a completely neutral fashion [27]. If this were the case for the chalcone synthase (*Chs*) and alcohol dehydrogenase (*Adh*) loci, their synonymous sites would be evolving more slowly than LTR-RT fossils, and the dating method described above would systematically overestimate the ages of their insertion events.

### *Athila *and *Gypsy*-like elements were more active in the past

The age distribution of complete *Copia*-like elements appears to show a recent burst of activity (Figure 1), but I provide evidence (below) that the excess of very young elements is the result of the rapid (relative to Metaviridae insertions) elimination of these elements from the genome. In contrast, the age distributions of complete *Athila *and *Gypsy*-like insertions have peaks between 1 and 2 Myr ago (Figure 1). Moreover, whereas there are 34 *Copia*-like insertions with their intra-element LTRs identical in sequence, only four such *Athila *and three such *Gypsy*-like insertions are present. These results indicate that levels of transpositional activity of *Athila *and *Gypsy*-like elements have declined since their peak between 1 and 2 Myr ago.

### Physical distribution

**Figure 2:**
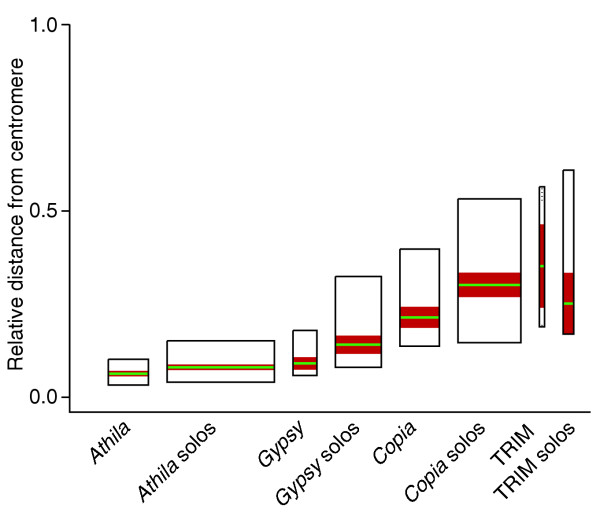
Differential pericentromeric clustering of complete elements and solo-LTRs along the 10 chromosome arms combined. The vertical axis measures distance from the centromere, divided by the length of the chromosome arm in which a given element is inserted: the value of 0.0 corresponds to the position of the centromeres and 1.0 to telomeres. Box heights indicate the inter-quartile range and widths are proportional to sample size; red bands represent 95% confidence limits on the median; and the green stripe marks the median value of each sample. Coordinates for the approximate centers of the centromeres on the chromosome sequences were set at 14.70 Mbp for chromosome I (total length 30.14 Mbp), at 3.70 Mbp for II (19.85 Mbp), at 13.70 Mbp for III (23.76 Mbp), at 3.10 Mbp for IV (17.79 Mbp), and at 11.80 Mbp for V (26.99 Mbp).

The chromosomal distribution of retrotransposons (and other TEs) in *A. thaliana *has been known to be non-random and dominated by a high concentration of elements in the heterochromatic pericentromeric regions [14]. However, this study has revealed significant differences in the chromosomal locations of the LTR-RT superfamilies. I have analyzed the distribution of complete elements and of solo-LTRs in each superfamily along all the chromosome arms combined, relative to the position of the centromeres (that is, the distribution of the distances between each insertion and the centromere, divided by the length of the respective arm), with results summarized in Figure 2.

*Athila *elements are almost exclusively inserted in the pericentromeric regions, and the other superfamilies in significantly and progressively less proximal regions of the chromosome arms (Wilcoxon rank sum tests: *Athila *more proximal than the *Gypsy*-like, *p *< 0.0001; *Gypsy*-like more proximal than *Copia*-like, *p *< 0.0001; complete *Copia*-like elements more proximal than complete TRIM elements, *p *< 0.05; there is no difference between *Copia*-like and TRIM solo-LTRs). Furthermore, except for TRIM, within each superfamily the solo-LTRs are significantly more distal than the complete elements (Wilcoxon rank sum tests, *p *< 0.001), suggesting that formation of solo-LTRs is more likely to occur in distal regions. The distribution of complete TRIM elements relative to the centromere is not significantly different from random (goodness-of-fit test, χ^2 ^= 4.22, df = 3, *p *> 0.2), although sample size is small, while their solo-LTRs are significantly clustered (goodness-of-fit test, χ^2 ^= 10.70, df = 3, *p *< 0.02).

### Accumulation in proximal regions by distinct evolutionary mechanisms: purifying selection and insertion bias

**Figure 3:**
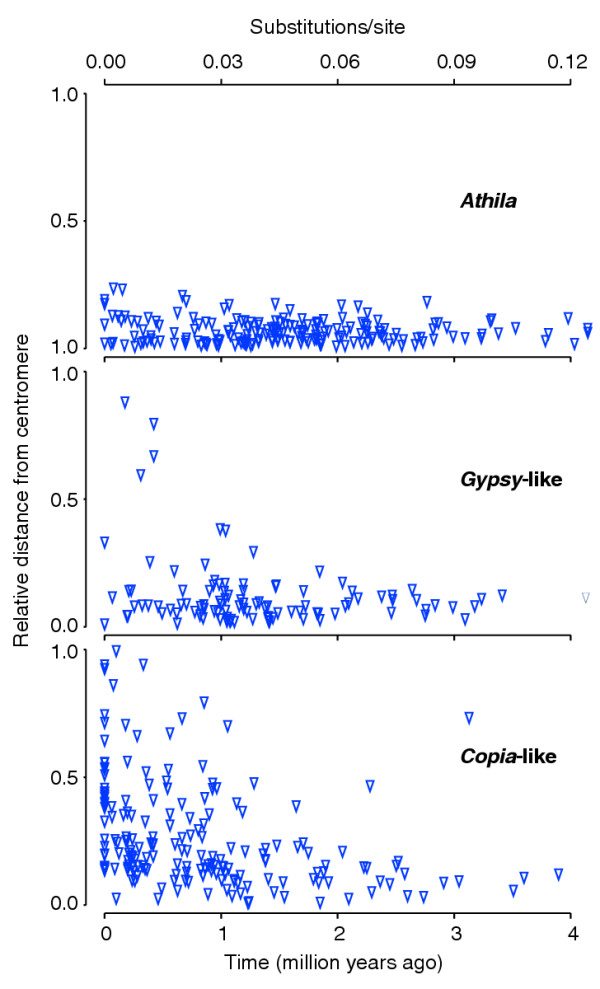
Relationship between age and physical distributions of complete elements. Insertions into the short arms of chromosomes II and IV were excluded for clarity. These arms contain extensive heterochromatin away from the centromeres, in nucleolar-organizing regions that juxtapose their telomeres, and in a knob [14]. In addition, their short length implies that the pericentromeric heterochromatin, which spans around 1-1.5 Mbp in each arm [68], corresponds to a substantially higher fraction of their total length than in the other eight arms.

The results above indicate that the older a superfamily is, the more its elements are concentrated in the proximal regions. This suggests that insertions into proximal (heterochromatic) regions are more likely to persist for longer periods of time. This interpretation assumes that the neutral mutation rate is the same for both the distal (euchromatic) and proximal (heterochromatic) portions of the genome. Intra-genomic variation in the per-replication mutation rate has been reported between the two sex chromosomes of a flowering plant [28] (although the difference could not be explained their different degree of DNA methylation, a feature often associated with heterochromatin). Given that the dating method used here is based on neutral sequence divergence (between intra-element LTRs), a higher mutation rate in heterochromatin in *A. thaliana *would affect age comparisons among different groups of elements, as they show different degrees of clustering into the pericentromeric heterochromatin. However, older estimates for the age of heterochromatic elements are consistent with the hypothesis that heterochromatin is a 'safe haven' where TE insertions persist for longer periods of time. Here I show that the mechanisms that led to the accumulation of LTR-RTs in proximal regions are distinct for different groups: elements of the youngest superfamily (*Copia*-like) insert randomly into the genome (relative to the location of the pericentromeric heterochromatin), but there is negative selection (on the host genome) against their insertions in euchromatin; elements of the older superfamilies (*Athila*, *Gypsy*-like) preferentially insert into the pericentromeric regions. These distinct mechanisms become apparent when temporal and spatial data are combined (Figure 3), and the chromosomal distribution of young elements compared with the distribution of older elements (within each superfamily).

For complete *Copia*-like elements there is a highly significant negative correlation between relative distance from the centromere and age of the insertions (Spearman rank correlation, ρ = -0.39, *p *< 0.0001). Furthermore, the distribution along the chromosome arms of 34 *Copia*-like insertions with no divergence between their intra-element LTRs is not significantly different from random (goodness-of-fit test, χ^2 ^= 3.12, df = 3, *p *> 0.3). This is evidence that *Copia-*like elements integrate randomly relative to the location of the centromeres, but tend to get eliminated from distal, and passively accumulate in proximal regions.

The average time to fixation (*t*) for a neutral allele is given by *t *= 4*N*_*e*_, where *N*_*e *_is the effective population size. For *A. thaliana t *can be estimated using an average of estimates of nucleotide diversity (*θ*) for 8 different *A. thaliana *genes, *θ* = 9 × 10^-3 ^[29], and the synonymous rate of substitution per site per generation, *μ* = 1.5 × 10^-8 ^[25]. *t *= 2*θ*/*μ*, yielding an estimate of *t *≈ 1.2 Myr. This value for *t *is consistent with an independent estimate that placed the time since the divergence between *A. thaliana *and *A. lyrata *between 3.45*t *and 5.6*t *[30]. Given that 75% of all complete *Copia*-like insertions are younger than 1.2 Myr, most of them are likely to be polymorphic. Taken together with the highly significant negative correlation between age and distance from the pericentromeric regions, these results indicate that complete *Copia*-like insertions are less likely to get fixed in the distal, euchromatic portions of the chromosome arms than in the pericentromeric heterochromatin.

In contrast, there is no correlation between age and relative distance from centromeres for complete *Athila *elements (Spearman rank correlation, ρ = 0.01, *p *= 0.9), as both young and old insertions are found only in proximal regions (Figure 3), compartmentalized into the pericentromeric heterochromatin. This strongly suggests that elements in the superfamily have evolved to preferentially target the pericentromeric heterochromatin, and their genomic distribution, unlike that of *Copia*-like elements, is not the result of passive accumulation therein. Only if *Athila *insertions were much more deleterious than *Copia*-like ones, so that they would be very rapidly removed by purifying selection, could passive accumulation be the case.

*Gypsy*-like insertions display a similar pattern to *Athila*. Even though there is for complete elements a significant, negative correlation between relative distance from centromeres and age, this is due to an excess of recent insertions near the telomere of the short arm of chromosome II (data not shown). If the arm is excluded from the analysis there is no significant correlation (Spearman rank correlation, ρ = -0.09, *p *> 0.3). This suggests that for the *Gypsy*-like also there is an insertional bias towards proximal regions. This bias is not as strong as for *Athila*, as complete *Gypsy*-like insertions are not exclusively found around the centromeres, and they cluster (to a much lesser extent) in at least one other heterochromatic region (the telomere of the short arm of chromosome II). Included in the *Gypsy*-like 'superfamily' is a clade of elements, known as *Tat*, which is a sister group to *Athila *to the exclusion of the remaining *Gypsy*-like elements [31]. The age and physical distribution of *Tat *does not differ from those of the remaining *Gypsy*-like elements (Wilcoxon rank-sum tests, *p *> 0.4); *Tat *show insertion bias towards the pericentromeric regions, but again to a lesser degree than *Athila*.

### Half-life of complete *Copia*-like insertions

**Figure 4:**
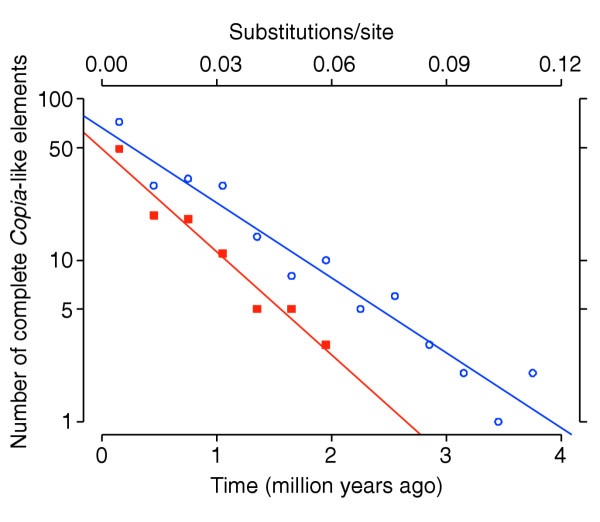
Loss of complete *Copia*-like elements. The half-life of complete *Copia*-like elements throughout the whole genome (log-transformed counts marked by blue circles, blue regression line) is estimated as around 650,000 ± 50,000 years. Complete insertions outside the proximal regions (red squares, red regression line) are lost more rapidly, with a half-life estimated as around 470,000 ± 50,000 years.

Given that *Copia*-like elements have been active until recently but tend to be eliminated by purifying selection, their age distribution (Figure 1, bottom) reflects the process of origin and loss of complete elements, when averaged over evolutionary time scales (and over all Pseudoviridae lineages). If this is assumed to be a steady-state process, it can be modeled by the survivorship function: *N*(*K*) = *N*_*o*_e^-*aK*^, where *N(K) *is the number of elements observed with intra-element LTR divergence *K*, and *N*_*o *_and *a *are constants to be fitted. The rate of elimination can then be estimated by linear regression of the log-transformed data (the half-life of insertions is given by ln2/*a*). Figure 4 shows the fit for all complete *Copia*-like insertions (*R*^2 ^= 0.94), and for complete insertions outside the proximal regions (i.e. with relative distance from centromeres >0.2; *R*^2 ^= 0.95). Complete *Copia-*like elements are eliminated from the genome with a half-life of 648,000 years (*SE *= 48,000 years). Insertions exclusively outside the proximal (heterochromatic) regions are lost more rapidly, with a half-life of 472,000 years (*SE *= 46,000 years).

## Discussion

The results above indicate that within a single genome, distinct evolutionary mechanisms can lead to the non-random distribution of retrotransposons, as in *A. thaliana *the accumulation of insertions in the pericentromeric heterochromatin is the result of both insertion bias (for Metaviridae elements) and a lower probability of fixation in euchromatin (Pseudoviridae).

It has recently been shown that most TE lineages in *A. thaliana *were already present in its common ancestor with *Brassica oleracea *(the two species diverged around 15-20 Myr ago), and that copy numbers are generally higher in *B. oleracea *[32]. The authors suggested that differential amplification of TEs between *A. thaliana *and *B. oleracea *was responsible for the larger genome of the latter. Here I have shown that the major LTR-RT families have been active in *A. thaliana *since its divergence from its closest relatives, such as *A. lyrata*. The transpositional activity of *Metaviridae *elements has declined relative to its level between 1 and 2 Myr ago, perhaps suggesting that the host genome has more efficiently suppressed their transposition since. However, Pseudoviridae (*Copia*-like) elements in *A. thaliana *have been subject to constant turnover. They have been recently active and show no insertion bias, and I estimate that the half-life of a complete element inserted in the euchromatic (non-coding) regions of the chromosome arms as around 470,000 years. Most of these *Pseudoviridae *insertions are lost before they reach fixation, and the half-life estimate provides a measure of the pace at which natural selection on the host constrains the genomic distribution and copy number of Pseudoviridae insertions. Turnover of Pseudoviridae insertions, in contrast to the longer persistence of Metaviridae elements that have declined in activity, is consistent with the higher sequence diversity among the Pseudoviridae than the Metaviridae in *A. thaliana *(107 Repbase update (RU) 'families' represented in 215 complete Pseudoviridaeelements, 25 RU 'families' in 349 complete Metaviridae elements, where 'families' were defined on the basis of sequence divergence); frequent reverse transcription during transposition would be likely to lead to faster evolution than that generated by the host genome DNA polymerase error rate on chromosomal insertions.

The lower probability of fixation in euchromatin relative to heterochromatin implies that insertions into euchromatin are more deleterious to the host (and perhaps that purifying selection is less efficient in heterochromatin due to a much reduced rate of recombination). TE density in the *A. thaliana *genome does not correlate with local recombination rate [33], providing some evidence against the ectopic recombination model for the deleterious effects of insertions (if the occurrence of ectopic and meiotic recombination positively correlate). Consistent with my results, the same study supports a model of purifying selection against insertions in intergenic DNA, by inferring that they are less likely to be found near genes [33].

As an alternative to selection, a neutral mutational process that deletes (part of the) insertions could in principle be driving the distribution of *Copia*-like elements, if such a process occurred more often in the euchromatic than in the pericentromeric regions of the genome, and if it were frequent enough. One mechanism that removes LTR-RT DNA from the genome is solo-LTR formation via unequal homologous recombination between intra-element LTRs. However, this mechanism cannot be the driving force shaping the distribution of complete *Copia*-like elements because *Copia*-like solo-LTRs are also non-randomly distributed and clustered in proximal regions (goodness-of-fit test: χ^2 ^= 13.71, df = 3, *p *< 0.005). *Copia*-like solo-LTRs are either eliminated faster from distal than proximal regions, like complete elements, or solo-LTR formation on average occurs more slowly than extinction for euchromatic insertions. Despite clustering around the centromeres, *Copia*-like solo-LTRs are significantly more dispersed than complete elements. This suggests that solo-LTRs do form before extinction for distal insertions, but are probably less efficiently eliminated (possibly because they are less deleterious to the host genome) than complete elements. Another known mechanism of (general) DNA loss operates via small deletions due to illegitimate recombination (between short repeats); this has been shown to occur in the *A. thaliana *genome by an analysis of internal deletions in LTR-RTs [26]. In *Drosophila*, rates of spontaneous deletions in euchromatin and heterochromatin do not seem to differ [34]. In *A. thaliana *the relative rates between the two chromatin domains are unknown, but fragmented (that is, neither solo-LTR nor complete) *Copia*-like insertions are as clustered around the centromeres as complete ones (goodness-of-fit test: χ^2 ^= 80.36, df = 3, *p *< 0.0001). Therefore small, spontaneous deletions cannot account for the genomic distribution of complete elements. Larger deletions (that remove the entire LTR-RT sequence) occurring primarily in euchromatin would be necessary to explain the observed accumulation pattern; if such a mechanism existed it would be an important force for genome size contraction. As there is no evidence for such mechanism, and given that I estimate that the half-life of (complete) insertions to be less than half the average time to fixation for a neutral allele, a lower probability of fixation in euchromatin relative to the pericentromeric heterochromatin is more likely to be driving the genomic distribution of Pseudoviridae elements.

It is interesting to note that the integrase proteins encoded by LTR-RTs differ between the Pseudoviridae and the Metaviridae in their carboxy-terminal domains, as they have different characteristic motifs [35,36]. This is the least conserved domain of integrase, and has been implicated in the insertion preferences of certain families of LTR-RTs in different organisms [37]. Examples of families of LTR-RTs whose integrase carboxy termini have been shown to interact with chromatin are known for both the Metaviridae [36] and the Pseudoviridae [38], and manipulation of this domain to engineer the targeting specificity of LTR-RTs has also been achieved [39]. *Athila *elements have been known to be present in the *A. thaliana *core centromeric arrays of the 180-bp satellite repeats and are abundant in pericentromeric heterochromatin [40,41]. In this study I have shown that in contrast with the passive accumulation of *Copia*-like elements, the striking compartmentalization of both recent and older *Athila *insertions in the pericentromeric heterochromatin indicates that these elements actively target those regions, and represents an example of a group of retrotransposons that have evolved to colonize a particular 'genomic niche'. Passive accumulation could not explain the distribution of *Athila *insertions unless they were generally much more deleterious to their host than *Copia*-like ones. Given the absence of complete *Athila *insertions from euchromatin, any one insertion would have to be so deleterious as to be almost immediately eliminated by purifying selection, even from intergenic DNA. Rather, it is likely that *Athila *elements preferentially insert into the pericentromeric heterochromatin and it is possible that this group of elements has been co-opted to play a part in centromere function. There is some evidence that such hypothetical role cannot be that of *cis*-acting sequences [42], but it could be a structural one. Studies on the appearance of neocentromeres [43-45] point to some degree of epigenetic regulation and function of centromeres via chromatin structuring. Although centromeric sequences are not conserved among plants [46], centromere-specific families of LTR-RTs seem to be common, as they have been found in cereals [47-51], chickpeas [52] and *A. thaliana *[40].

Both purifying selection (at the host level) against insertions (in euchromatin) and a decline in transpositional activity (of Metaviridae elements) appear to have limited the recent contribution of retrotransposon DNA to genome size expansion in *A. thaliana*. The rapid and recent genome evolution inferred for *A. thaliana *may be a feature common to other higher eukaryotes, in particular those with compact genomes. High turnover of TE insertions in euchromatin also occurs in *Drosophila *and pufferfish [53], for example, and accumulation of TEs into heterochromatin in those genomes may also, as in *A. thaliana*, be due to diverse evolutionary mechanisms.

## Materials and methods

A methodology was developed for the automated mining of sequence data to retrieve the sequence and chromosomal location of genomic 'fossils' of LTR-RTs, identifying complete elements and solo-LTRs among the retrieved sequence fragments, and estimating the age of the insertion events that gave origin to these elements. This methodology was applied to the genome sequence of *A. thaliana*.

### Molecular paleontology of LTR-retrotransposons

Sequences of the organellar and the five nuclear chromosomes (version 200303) were obtained from the Munich Information Center for Protein Sequences (MIPS) [54]. Computational mining for LTR-RT fragments in the *A. thaliana *genome (around 116 Mbp of available sequence) was performed using sequence-similarity search algorithms [55] against a library of representative sequences of LTR-RTs. This reference library was compiled by extracting from Repbase update [56,57] sequences of the LTRs and internal region (IR) of known *A. thaliana *'families' of LTR-RTs. The programs RepeatMasker [58] and WU-BLAST [59] were used to search the whole genomic sequence (initially divided into 50 kbp chunks) and obtain the precise coordinates of chromosomal segments homologous to (a part of) the LTR or IR of library elements. The datasets of chromosomal coordinates of the complete LTR-RTs and solo LTRs identified are available as Additional data files 1 and 3.

'Families' of LTR-retrotransposons (as classified in Repbase update) are present in low copy numbers; therefore, for the purpose of this analysis they were grouped into three 'superfamilies': *Athila*, *Gypsy*-like (all 'families' belonging to the Metaviridae, excluding *Athila*), and *Copia*-like (all 'families' belonging to the Pseudoviridae). The Metaviridae was split into two groups (*Athila *and *Gypsy*-like), as initial mining of the *A. thaliana *genome revealed that *Athila *elements have been particularly successful in colonizing it. Their copy number is roughly double the number of all other members of the Metaviridae, and higher than the total of all Pseudoviridae elements. *Athila *form a clade and are retroviral-like elements that are likely to have an *envelope *(*env*) gene [60]. Most of the *Copia*- and *Gypsy-*like elements are typical LTR-RTs, although one of the *Copia*-like 'families' (*metaI*) comprises non-autonomous elements [22] and a few others (*endovir1 *[61], *atcopia41-43 *[22]) are retroviral-like, featuring a putative *env *gene. A fourth 'superfamily' was used to include TRIMs. These are short, non-autonomous elements that feature LTRs but no coding genes and cannot currently be classified into either the Pseudoviridae or the Metaviridae; they are described in [62].

The four superfamilies comprise the following 'families'. ***Athila ***(10 families): *athila2 - 5*, *athila4A - D*, *athila6A*, *athila7*, *athila8A *and *B*; ***Gypsy*-like **(15 families): *atgagpol1*, *atgp2 *and *3*, *atgp2N*, *atgp5 - 10*, *atgp9B*, *atlantys1 - 3*, *tat1*; ***Copia*-like **(107 families): *atcopia1 - 97*, *atcopia8A *and *B*, *atcopia18A*, *atcopia32B*, *atcopia38A *and *B*, *atcopia65A*, *endovir1*, *TA1-2*, *meta1*; **TRIM **(3 families): *katydid-At1*, *katydid-At2*, *katydid-At3*.

### Identification of complete elements and solo-LTRs

A *Perl *script, LTR_MINER (available on request), was written to parse all the chromosomal LTR-RT fragments reported by RepeatMasker (WU-BLAST hits of similarity to reference sequences) and identify complete elements and solo-LTRs. LTR_MINER performs the pattern-recognition function of assembling hits that originated from single LTR-RT insertion events. The algorithm involves: 'defragmentation' of LTR hits. If a chromosomal LTR fossil contains insertions/deletions (indels) relative to the most similar library sequence, it may be reported as multiple hits (fragments). Defragmentation is the identification of multiple hits that correspond to the same LTR. Parameters were set so that LTR hits were defragmented only when they were separated by no more than 550 bp, belonged to the same family, had the same orientation on the chromosome, and their combined length did not exceed the length of the corresponding family reference sequence by more than 20 bp.

### Identification of 'complete' elements

An intact LTR-RT insertion consists of at least three hits: LTR-IR-LTR (an IR from a single element insertion may also yield multiple hits). After LTR defragmentation, LTR_MINER searches for contiguous patterns of *LTR*, *IR*, *LTR*. In order to check whether the pattern could be straddling a nested insertion of the same family, the search is then recursively extended from each end of the pattern for further contiguous hits to an IR and a LTR (of same family and orientation). The two LTRs of the innermost pattern are classified as a pair of intra-element LTRs.

### Identification of 'interrupted' elements: fossil elements containing insertions between the two LTRs

LTR_MINER also identifies such elements provided an IR is present between the LTRs. A maximum pairing distance between LTRs was set at 30 kb.

### Identification of 'solo-LTRs'

LTR_MINER was set to classify a LTR fragment as a solo-LTR if no other LTR or IR (of same family and orientation) is present within a 5 kbp radius from the fragment's ends. The aim was the identification of elements resulting from deletion (of the IR and one LTR) events via homologous recombination between intra-element LTRs, and not to classify as solo-LTRs sequences that are separated from IRs because of insertions.

### Dating of insertion events

Nucleotide sequence divergence between pairs of intra-element LTRs was used as a molecular clock, as these pairs are identical at the time of insertion [63]. All mined pairs of intra-element LTR sequences were aligned using ClustalW [64] (with Pwgapopen = 5.0, Pwgapext = 1.0). To ensure correct alignment of any sequences with large indels, pairwise LTR alignments were position-anchored relative to reference sequences: if a chromosomal LTR fossil consisted of multiple hits (of similarity to segments of the reference sequence) then the intervening chromosomal sequence between such hits was replaced by a number of gaps, equal to the length of the region separating the corresponding segments in the reference. The number of nucleotide substitutions per site (*K*) between each intra-element LTR pair was then estimated using Kimura's two-parameter model [65]. To reduce sampling bias towards younger elements, elements with truncated LTRs were included in the analysis (provided both LTRs are present), as intact elements are likely to be younger than elements that have accumulated indels.

Alignments with fewer than 80 nucleotides were discarded. As CLUSTAL-W alignments could be poor if LTR sequences were only partially overlapping, for all LTR pairs with *K *greater than 0.2 they were inspected by eye and manually edited if necessary (and *K *then recalculated). Estimates of the ages of insertion were obtained by using the equation *t = K/2r*, where *t *is the age, and *r *is nucleotide substitution rate for the host genome DNA polymerase. The value of 1.5 × 10^-8 ^substitutions per site per year was used for *r *(1.0 <*r *< 2.1 × 10^-8 ^95% confidence interval), estimated in [25] for the synonymous substitution rate in the *Chs *and *Adh *loci in *Arabidopsis*/*Arabis *species.

Finally, if recombination between LTRs from different insertions had occurred frequently, the dating method above would be invalid for obtaining the age profiles of different families. To detect possible recombination events, multiple alignments of all LTRs (including solos) of certain families were generated using BLASTALIGN [66], a program that can handle datasets that may contain large indels. Neighbor-joining trees of the LTR sequences were then constructed using PAUP* 4.0b10 [67] with the HKY85 model, to check whether intra-element LTR pairs clustered.

## Additional data files

The following additional data files are available with the online version of this article. Additional data file 1 contains the entire dataset of chromosomal coordinates and ages of complete LTR-retrotransposons in *A. thaliana*. Additional data file 2 describes the data fields in Additional data file 1. Additional data file 3 contains the entire dataset of chromosomal coordinates of solo-LTRs in *A. thaliana*. Additional data file 4 describes the data fields in Additional data file 3. Additional data file 5 contains the *Perl *script LTR_MINER, used to de-fragment sequence similarity hits to LTR-retrotransposons, and identify complete and solo-LTR elements. Additional data file 6 describes the utility and usage of the *Perl *script in Additional data file 5. Additional data file 7 contains the *Perl *script used in conjunction with LTR_MINER, used to divide long sequences into smaller chunks labeled by their coordinate range. Additional file data 8 describes the usage of the *Perl *script in Additional data file 7.

## Supplementary Material

Additional data file 1 The entire dataset of chromosomal coordinates and ages of complete LTR-retrotransposons in *A. thaliana* Click here for additional data file

Additional data file 2 A file describing the data fields in Additional data file 1 Click here for additional data file

Additional data file 3 The entire dataset of chromosomal coordinates of solo-LTRs in *A. thaliana* Click here for additional data file

Additional data file 4 A file describing the data fields in Additional data file 3 Click here for additional data file

Additional data file 5 The *Perl *script LTR_MINER, used to de-fragment sequence similarity hits to LTR-retrotransposons, and identify complete and solo-LTR elements Click here for additional data file

Additional data file 6 A file describing the utility and usage of the *Perl *script in Additional data file 5 Click here for additional data file

Additional data file 7 The *Perl *script used in conjunction with LTR_MINER, used to divide long sequences into smaller chunks labeled by their coordinate range Click here for additional data file

Additional data file 8 A file describing the utility and usage of the *Perl *script in Additional data file 7 Click here for additional data file

## Figures and Tables

**Table 1:** Relative abundance of LTR-retrotransposons in *Arabidopsis thaliana*

Superfamily	Percentage of genome*	Number of complete elements^†^	Percentage DNA in complete elements^†^	Number of solo-LTRs
*Athila*	2.73 %	219	50 %	586
*Gypsy*-like	1.32 %	130	53 %	250
*Copia*-like	1.39 %	215	63 %	343
TRIM	0.15 %	28	53 %	58
Total	5.60 %	592	54 %	1,237

*The '% of genome' includes all LTR-RT sequences (in the nuclear genome) for each superfamily, rather than just complete and solo-LTR elements. Fragments of LTR-RTs were also found in the mitochondrial (2.74%) and chloroplast (0.05%) genomes. ^†^Elements containing indels were included as complete elements provided they retain a substantial part of both their LTRs.

## References

[B1] Kapitonov VV, Jurka J (2003). Molecular paleontology of transposable elements in the *Drosophila melanogaster *genome.. Proc Natl Acad Sci USA.

[B2] Smit AF (1999). Interspersed repeats and other mementos of transposable elements in mammalian genomes.. Curr Opin Genet Dev.

[B3] SanMiguel P, Gaut BS, Tikhonov A, Nakajima Y, Bennetzen JL (1998). The paleontology of intergene retrotransposons of maize.. Nat Genet.

[B4] Kazazian HH (2004). Mobile elements: drivers of genome evolution.. Science.

[B5] Thomas C (1971). The genetic organization of chromosomes.. Annu Rev Genet.

[B6] Kidwell MG (2002). Transposable elements and the evolution of genome size in eukaryotes.. Genetica.

[B7] Wendel JF (2000). Genome evolution in polyploids.. Plant Mol Biol.

[B8] Kalendar R, Tanskanen J, Immonen S, Nevo E, Schulman AH (2000). Genome evolution of wild barley (*Hordeum spontaneum*) by *BARE-1 *retrotransposon dynamics in response to sharp microclimatic divergence.. Proc Natl Acad Sci USA.

[B9] Vicient CM, Suoniemi A, Anamthawat-Jonsson K, Tanskanen J, Beharav A, Nevo E, Schulman AH (1999). Retrotransposon *BARE-1 *and its role in genome evolution in the genus *Hordeum*.. Plant Cell.

[B10] Kumar A, Bennetzen JL (1999). Plant retrotransposons.. Annu Rev Genet.

[B11] Dasilva C, Hadji H, Ozouf-Costaz C, Nicaud S, Jaillon O, Weissenbach J, Crollius HR (2002). Remarkable compartmentalization of transposable elements and pseudogenes in the heterochromatin of the *Tetraodon nigroviridis *genome.. Proc Natl Acad Sci USA.

[B12] Bartolome C, Maside X, Charlesworth B (2002). On the abundance and distribution of transposable elements in the genome of *Drosophila melanogaster*.. Mol Biol Evol.

[B13] Kidwell MG, Lisch DR (2001). Perspective: transposable elements, parasitic DNA, and genome evolution.. Evolution Int J Org Evolution.

[B14] The *Arabidopsis *Genome Initiative (2000). Analysis of the genome sequence of the flowering plant *Arabidopsis thaliana*.. Nature.

[B15] Orgel LE, Crick FH (1980). Selfish DNA: the ultimate parasite.. Nature.

[B16] Doolittle WF, Sapienza C (1980). Selfish genes, the phenotype paradigm and genome evolution.. Nature.

[B17] Yoder JA, Walsh CP, Bestor TH (1997). Cytosine methylation and the ecology of intragenomic parasites.. Trends Genet.

[B18] Langley CH, Montgomery E, Hudson R, Kaplan N, Charlesworth B (1988). On the role of unequal exchange in the containment of transposable element copy number.. Genet Res.

[B19] Biemont C, Tsitrone A, Vieira C, Hoogland C (1997). Transposable element distribution in *Drosophila*.. Genetics.

[B20] Nuzhdin SV, Pasyukova EG, Mackay TF (1996). Positive association between *copia *transposition rate and copy number in *Drosophila melanogaster*.. Proc R Soc Lond B Biol Sci.

[B21] Dimitri P, Junakovic N (1999). Revising the selfish DNA hypothesis: new evidence on accumulation of transposable elements in heterochromatin.. Trends Genet.

[B22] Kapitonov VV, Jurka J (1999). Molecular paleontology of transposable elements from *Arabidopsis thaliana*.. Genetica.

[B23] Le QH, Wright S, Yu Z, Bureau T (2000). Transposon diversity in *Arabidopsis thaliana*.. Proc Natl Acad Sci USA.

[B24] Bennett MD, Leitch IJ, Price HJ, Johnston JS (2003). Comparisons with *Caenorhabditis *(approximately 100 Mb) and *Drosophila *(approximately 175 Mb) using flow cytometry show genome size in Arabidopsis to be approximately 157 Mb and thus approximately 25% larger than the *Arabidopsis *genome initiative estimate of approximately 125 Mb.. Ann Bot (Lond).

[B25] Koch MA, Haubold B, Mitchell-Olds T (2000). Comparative evolutionary analysis of chalcone synthase and alcohol dehydrogenase loci in *Arabidopsis*, *Arabis*, and related genera (Brassicaceae).. Mol Biol Evol.

[B26] Devos KM, Brown JK, Bennetzen JL (2002). Genome size reduction through illegitimate recombination counteracts genome expansion in *Arabidopsis*.. Genome Res.

[B27] Duret L, Mouchiroud D (1999). Expression pattern and, surprisingly, gene length shape codon usage in *Caenorhabditis*, *Drosophila*, and *Arabidopsis*.. Proc Natl Acad Sci USA.

[B28] Filatov DA, Charlesworth D (2002). Substitution rates in the X- and Y-linked genes of the plants, *Silene latifolia *and *S. dioica*.. Mol Biol Evol.

[B29] Tian D, Araki H, Stahl E, Bergelson J, Kreitman M (2002). Signature of balancing selection in *Arabidopsis*.. Proc Natl Acad Sci USA.

[B30] Bustamante CD, Nielsen R, Sawyer SA, Olsen KM, Purugganan MD, Hartl DL (2002). The cost of inbreeding in *Arabidopsis*.. Nature.

[B31] Peterson-Burch BD, Nettleton D, Voytas DF (2004). Genomic neighborhoods and *Arabidopsis *retrotransposons: Genome sequence analysis reveals a role for targeted integration in the distribution of *Athila *and *Tat *elements.. Genome Biol.

[B32] Zhang X, Wessler SR (2004). Genome-wide comparative analysis of the transposable elements in the related species *Arabidopsis thaliana *and *Brassica oleracea*.. Proc Natl Acad Sci USA.

[B33] Wright SI, Agrawal N, Bureau TE (2003). Effects of recombination rate and gene density on transposable element distributions in *Arabidopsis thaliana*.. Genome Res.

[B34] Blumenstiel JP, Hartl DL, Lozovsky ER (2002). Patterns of insertion and deletion in contrasting chromatin domains.. Mol Biol Evol.

[B35] Peterson-Burch BD, Voytas DF (2002). Genes of the Pseudoviridae (*Ty1/copia *retrotransposons).. Mol Biol Evol.

[B36] Malik HS, Eickbush TH (1999). Modular evolution of the integrase domain in the *Ty3/Gypsy *class of LTR retrotransposons.. J Virol.

[B37] Sandmeyer S (2003). Integration by design.. Proc Natl Acad Sci USA.

[B38] Xie W, Gai X, Zhu Y, Zappulla DC, Sternglanz R, Voytas DF (2001). Targeting of the yeast *Ty5 *retrotransposon to silent chromatin is mediated by interactions between integrase and Sir4p.. Mol Cell Biol.

[B39] Zhu Y, Dai J, Fuerst PG, Voytas DF (2003). Controlling integration specificity of a yeast retrotransposon.. Proc Natl Acad Sci USA.

[B40] Pelissier T, Tutois S, Tourmente S, Deragon JM, Picard G (1996). DNA regions flanking the major *Arabidopsis thaliana *satellite are principally enriched in *Athila *retroelement sequences.. Genetica.

[B41] Kumekawa N, Hosouchi T, Tsuruoka H, Kotani H (2000). The size and sequence organization of the centromeric region of *Arabidopsis thaliana *chromosome 5.. DNA Res.

[B42] Nagaki K, Talbert PB, Zhong CX, Dawe RK, Henikoff S, Jiang J (2003). Chromatin immunoprecipitation reveals that the 180-bp satellite repeat is the key functional DNA element of *Arabidopsis thaliana *centromeres.. Genetics.

[B43] du Sart D, Cancilla MR, Earle E, Mao JI, Saffery R, Tainton KM, Kalitsis P, Martyn J, Barry AE, Choo KH (1997). A functional neo-centromere formed through activation of a latent human centromere and consisting of non-alpha-satellite DNA.. Nat Genet.

[B44] Karpen GH, Allshire RC (1997). The case for epigenetic effects on centromere identity and function.. Trends Genet.

[B45] Williams BC, Murphy TD, Goldberg ML, Karpen GH (1998). Neocentromere activity of structurally acentric mini-chromosomes in *Drosophila*.. Nat Genet.

[B46] Richards EJ, Dawe RK (1998). Plant centromeres: structure and control.. Curr Opin Plant Biol.

[B47] Ananiev EV, Phillips RL, Rines HW (1998). Chromosome-specific molecular organization of maize (*Zea mays *L.) centromeric regions.. Proc Natl Acad Sci USA.

[B48] Vitte C, Panaud O (2003). Formation of Solo-LTRs through unequal homologous recombination counterbalances amplifications of LTR retrotransposons in rice *Oryza sativa *L.. Mol Biol Evol.

[B49] Langdon T, Seago C, Mende M, Leggett M, Thomas H, Forster JW, Jones RN, Jenkins G (2000). Retrotransposon evolution in diverse plant genomes.. Genetics.

[B50] Kumekawa N, Ohmido N, Fukui K, Ohtsubo E, Ohtsubo H (2001). A new *gypsy*-type retrotransposon, *RIRE7*: preferential insertion into the tandem repeat sequence TrsD in pericentromeric heterochromatin regions of rice chromosomes.. Mol Genet Genomics.

[B51] Mroczek RJ, Dawe RK (2003). Distribution of retroelements in centromeres and neocentromeres of maize.. Genetics.

[B52] Staginnus C, Winter P, Desel C, Schmidt T, Kahl G (1999). Molecular structure and chromosomal localization of major repetitive DNA families in the chickpea (*Cicer arietinum *L.) genome.. Plant Mol Biol.

[B53] Volff JN, Bouneau L, Ozouf-Costaz C, Fischer C (2003). Diversity of retrotransposable elements in compact pufferfish genomes.. Trends Genet.

[B54] FTP directory/cress. ftp://ftpmips.gsf.de/cress.

[B55] Altschul SF, Gish W, Miller W, Myers EW, Lipman DJ (1990). Basic local alignment search tool.. J Mol Biol.

[B56] Repbase update - Genetic Information Research Institute. http://www.girinst.org/Repbase_Update.html.

[B57] Jurka J (2000). Repbase update: a database and an electronic journal of repetitive elements.. Trends Genet.

[B58] RepeatMasker home page. http://www.repeatmasker.org.

[B59] WU-BLAST. http://blast.wustl.edu.

[B60] Wright DA, Voytas DF (1998). Potential retroviruses in plants: *Tat1 *is related to a group of *Arabidopsis thaliana Ty3/gypsy *retrotransposons that encode envelope-like proteins.. Genetics.

[B61] Peterson-Burch BD, Wright DA, Laten HM, Voytas DF (2000). Retroviruses in plants?. Trends Genet.

[B62] Witte CP, Le QH, Bureau T, Kumar A (2001). Terminal-repeat retrotransposons in miniature (TRIM) are involved in restructuring plant genomes.. Proc Natl Acad Sci USA.

[B63] Varmus H (1988). Retroviruses.. Science.

[B64] Thompson JD, Higgins DG, Gibson TJ (1994). CLUSTAL W: improving the sensitivity of progressive multiple sequence alignment through sequence weighting, position-specific gap penalties and weight matrix choice.. Nucleic Acids Res.

[B65] Kimura M (1980). A simple method for estimating evolutionary rates of base substitutions through comparative studies of nucleotide sequences.. J Mol Evol.

[B66] Belshaw R, Katzourakis A (2004). *BlastAlign*: a program that uses *blast *to align problematic nucleotide sequences.. Bioinformatics.

[B67] Swofford D (1998). PAUP* Phylogenetic analysis using parsimony (*and other methods) Version 4.

[B68] Haupt W, Fischer TC, Winderl S, Fransz P, Torres-Ruiz RA (2001). The centromere1 (CEN1) region of *Arabidopsis thaliana*: architecture and functional impact of chromatin.. Plant J.

